# Determinants of loss to follow-up after eye-related emergency visits: A race-stratified analysis at a level I trauma center

**DOI:** 10.1371/journal.pone.0355731

**Published:** 2026-08-06

**Authors:** Sinan Ersan, Charles Zhang, Yousef Yousef, Nicholas Kemmis, Brendan Perreault, Ismet F. Vardar, Karen M. Allison, Andrew L. Reynolds, Margaret M. DeAngelis

**Affiliations:** 1 Jacobs School of Medicine and Biomedical Sciences, State University of New York at Buffalo, Buffalo, New York, United States of America; 2 Department of Ophthalmology, Bascom Palmer Eye Institute, University of Miami Miller School of Medicine, Miami, Florida, United States of America; 3 Department of Ophthalmology, Ross Eye Institute, University at Buffalo, Buffalo, New York, United States of America; 4 Rutgers University New Jersey Medical School, Newark, New York, United States of America; 5 Department of Ophthalmology, University of Rochester, Flaum Eye Institute, Rochester, New York, United States of America; 6 Department of Ophthalmology and Visual Sciences, University of Utah School of Medicine, The University of Utah, Salt Lake City, Utah, United States of America; 7 Department of Population Health Sciences, University of Utah School of Medicine, The University of Utah, Salt Lake City, Utah, United States of America; 8 Veterans Administration Western New York Healthcare System, Buffalo, New York, United States of America; 9 Department of Biochemistry, Jacobs School of Medicine and Biomedical Sciences, State University of New York, University at Buffalo, Buffalo, New York, United States of America; 10 Genetics, Genomics and Bioinformatics Graduate Program, Jacobs School of Medicine and Biomedical Sciences, State University of New York, University at Buffalo, Buffalo, New York, United States of America; 11 Neuroscience Graduate Program, Jacobs School of Medicine and Biomedical Sciences, State University of New York, University at Buffalo, Buffalo, New York, United States of America; Touro University California College of Pharmacy, UNITED STATES OF AMERICA

## Abstract

**Importance:**

Strict adherence to follow-up ophthalmic care after emergency department visits is critical not only for monitoring disease progression, assessing therapeutic response, and preventing avoidable complications, but also because ophthalmic examinations may provide one of the few opportunities to identify otherwise unrecognized systemic disease.

**Objective:**

To identify variables associated with loss to follow-up (LTFU) care after emergency department ophthalmology consultations, and to characterize similarities and differences between self-reported race.

**Design:**

Retrospective cohort study examining emergency department ophthalmology consultations between January 1^st^, 2019, and December 31^st^, 2021, at a level 1 trauma center serving Western New York, Northern Pennsylvania and Southern Ontario.

**Setting:**

Single center study at Erie County Medical Center in Buffalo, New York, USA.

**Participants:**

A total of 2323 ophthalmology consultations were analyzed.

**Main outcomes and measures:**

The primary outcome was the rate of LTFU, defined as failure to attend a scheduled outpatient appointment. Demographic, diagnostic, and socioeconomic variables were analyzed across all patients and stratified by self-reported racial group (White, African American/Black, and Other). Logistic regression analysis was performed.

**Results:**

Of the 1697/2323 (73.1%) patients that required an outpatient follow-up at The Ira G. Ross Eye Institute, 1003 (59.1%) identified as White, 489 (28.8%) identified as African American/Black and 205 (12.1%) identified as Other. The overall rate of LTFU was 41.8%, with no significant difference in follow-up rates by racial group on multivariable analysis. In the overall patient cohort, LTFU was significantly more common among patients residing in ZIP codes with lower rates of high school completion, those with a longer interval between the emergency department visit and the scheduled follow-up appointment, and those who presented with near-normal visual acuity (close to 20/20). Among White patients, glaucomatous and retinal diagnoses were associated with lower rates of LTFU. Older age, corneal diagnoses and orbital diagnoses were associated with lower rates of LTFU among African American/Black patients.

**Conclusions and Relevance:**

This study identified both shared and distinct factors associated with LTFU across racial groups. These findings provide a foundation for future studies investigating the mechanisms underlying follow-up adherence and may help inform efforts to improve outpatient follow-up.

## Introduction

Emergency departments (ED) across the United States provide care to over 130 million patients annually, including approximately 2 million visits for eye-related conditions [1–4]. The majority of these eye-related visits do not necessitate hospital admission and are followed closely in an outpatient setting by an ophthalmologist [4]. As a result, ensuring that patients attend these outpatient appointments is essential for improving overall health outcomes. Consistent follow-up visits allow healthcare providers to monitor disease progression, optimize treatment strategies, and ultimately reduce 30-day readmission rates which, on average, can cost over $16,000 in US hospital costs [5,6]. Furthermore, the eyes serve as a proxy for systemic health, as evidenced by conditions such as hypertension, diabetes mellitus and cardiovascular disease; thus, ophthalmic follow-up care can identify critical diagnostic and prognostic markers, ensuring prompt management and decreasing the overall burden on the healthcare system [7]. ED visits and the resulting follow-up appointments may be the only occasion in which these patients receive thorough eye examinations.

Despite the established importance of follow-up care, loss to follow-up (LTFU) rates after ED visits remain very high, ranging from 25% to 63% [8–16]. Notably, these rates appear to be different when the chief complaint involves an eye-related concern, implying that unique factors associated with eye-related ED visits may influence follow-up rates [10–14]. A prior study examining the rates of LTFU after eye-related ED visits showed that many factors, including younger age and Medicaid utilization, were associated with increased rates of LTFU [12]. However, there remains a paucity of research examining how additional socioeconomic, diagnostic and demographic variables influence follow-up rates after ED ophthalmology consultations across different racial groups [17–19]. The current study aims to address these gaps in knowledge to better equip physicians to reduce the high rates of LTFU and improve patient outcomes.

## Methods

This 3-year, single-institution retrospective cohort study evaluated patients seen at Erie County Medical Center (ECMC) emergency department in Buffalo, New York for eye-related emergencies. The study was approved by the State University of New York at Buffalo Institutional Review Board (IRB), which granted a waiver of informed consent. Furthermore, the current study was conducted in accordance with the tenets of the Declaration of Helsinki.

### Study setting and population

ECMC is a level 1 trauma center in Buffalo, New York, and serves as a safety-net hospital for Western New York, Northern Pennsylvania and Southern Ontario, providing care for many of the uninsured and underinsured population in the area [20]. In 2022, it was recognized as one of the top 50 hospitals in the nation for racial inclusivity, serving a very diverse population of patients [20]. For reference, 44.6% of the population in Buffalo, New York identify as White and 37.3% identify as African American/Black [21]. These figures differ markedly from the overall New York State population, in which 56.5% of residents identify as White and 15.6% identify as African American/Black [22].

Ophthalmologists at the Ira G. Ross Eye Institute (REI), which is under the auspices of the State University of New York at Buffalo, cover all eye-related consultations at ECMC. Per consultation guidelines, all patients seen at ECMC were scheduled for outpatient follow-up at the REI unless they met one of the following criteria: (1) the examination demonstrated benign ocular findings (e.g., dry eye syndrome, blepharitis) with visual acuity of 20/25 or better; or (2) the patient had an established eye care specialist who was willing and able to provide timely follow-up care, at which point care was transferred accordingly. Each patient who attended their follow-up appointment at the REI was seen free of charge, regardless of ability to pay, for up to two visits after their hospital stay. This policy was implemented to reduce the financial burden and potential deterrent associated with attending follow-up appointments as previously described [23]. The provision of free outpatient visits was thoroughly explained to all patients during the consultation, and again prior to discharge.

### Database creation

All ED ophthalmology consultations between January 1^st^, 2019 and December 31^st^, 2021 were ascertained and collected. The following data were captured for each visit: date of consultation, scheduled follow-up appointment (yes or no), attendance at follow-up appointment, date of birth, sex, home zip code, self-identified race (e.g., White, African American/Black, Other), primary language (English, other), diagnosis, visual acuity of the affected eye (Snellen), primary insurance (e.g., Medicaid, other), and prior medical history of standardized co-morbid diseases as previously defined in literature (e.g., acute myocardial infarction, congestive heart failure, cerebral vascular accident, etc) [24]. Visual acuity measurements were converted from Snellen notation to logarithm of the minimum angle of resolution (logMAR) units for statistical analysis. Non-numeric visual acuity measures, including counting fingers, hand motion, light perception, and no light perception, were assigned logMAR equivalents of 2.1, 2.4, 2.7, and 3.0, respectively. To satisfy the assumption of independent observations inherent to standard logistic regression models, only the initial encounter was included for patients with multiple visits during the study period.

The diagnosis during the eye-related consultation was recorded and categorized according to *The Wills Eye Manual: Office and Emergency Room Diagnosis and Treatment of Eye Disease, 8th edition* (WEM) [25] as previously described by our team [26]. Ten categories of disease were included: (1) trauma, (2) cornea, (3) conjunctiva/sclera/iris/external disease, (4) eyelid, (5) orbit, (6) pediatric, (7) glaucoma, (8) neuro-ophthalmology, (9) retina, and (10) uveitis. Diagnoses were categorized by broader groups rather than individual diagnoses due to nearly 200 conditions included in the current study, as separating them would significantly reduce analytical power [27]. A full list of diagnoses in each respective category is included in Supplementary File 1. We chose the WEM as our standardization tool because it is endorsed by the American Academy of Ophthalmology for delivering rigorous and robust clinical information to healthcare providers.

### Social determinants of health

Utilizing the 2021 American Census Bureau database, variables including income, percentage living below the poverty line, and percentage with a high school diploma in the respective ZIP codes were recorded. Although ZIP codes may not provide individual-level data, several studies have demonstrated the utility of using ZIP codes as a proxy for social determinants of health [28–34]. While other indices such as the neighborhood social vulnerability index (SVI) do exist, ZIP code-level analysis have several advantages in the current study [34]. First, utilization of ZIP codes allows for broader inclusion of patients and minimizes missing or incomplete data, thereby preserving study sample size and statistical power [34]. Second, while SVI combines multiple variables into a single composite measure that can be difficult to disentangle, ZIP code-level variables allow for clearer interpretation of associations and greater transparency by keeping socioeconomic factors such as income, poverty, and education status separate [34].

To assess potential barriers to transportation, the average distance (in miles) from the center of each zip code to the REI was determined using Google Maps. The REI is located approximately 3.4 miles from the ECMC ED, and several modes of public transportation are accessible to the public. The farther a patient resides from the Buffalo Niagara Medical Campus in downtown Buffalo, which includes the REI, the more difficult it may be to access public transportation. Therefore, transportation is arranged through ECMC for all patients that require transportation but cannot access or afford it.

### Statistical analysis

Baseline characteristics were initially analyzed and compared using two-tailed t-tests for continuous variables and Chi-square tests for categorical variables in the overall patient population as well as each racial group (White and African American/Black). Separate analyses were not performed for the “Other” race category because it included individuals from multiple racial groups as well as those who declined to disclose their race, making the group insufficiently representative for meaningful analysis. A Wald Z-test was used to compare outcomes from univariable logistic regression between White and African American/Black cohorts. Any variable identified with a *p* value < 0.15 on univariate analysis was then used to generate a multivariable logistic regression model in a bi-directional stepwise fashion based on Akaike Information Criterion for each dataset [35]. *P* values < 0.05 were considered statistically significant. Analyses were performed with R studio version 2022.04.2 + 764. The de-identified dataset supporting the findings of this study is available in Supplementary File 2.

## Results

A total of 2323 ophthalmology consultations were analyzed between Jan 2019 and Dec 2021. Of these, 1405 (60.5%) patients identified as White, 646 (27.8%) patients identified as African American/Black and 272 (11.7%) identified as Other.

**Fig 1:**
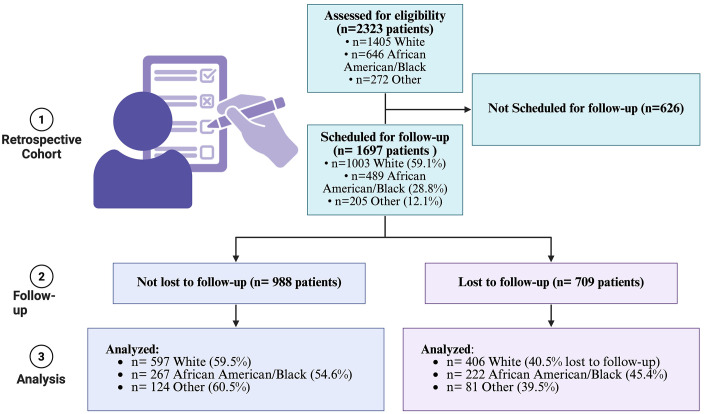
Flowchart of patient selection and inclusion for this retrospective cohort study, created using BioRender.com.

A total of 1697/2323 (73.1%) patients were scheduled for an outpatient follow-up appointment at the REI, of which 1003 identified as White (59.1%), 489 identified as African American/Black (28.8%) and 205 identified as Other (12.1%). Among the 1,697 patients with a scheduled follow-up appointment, 988 (58.2%) attended their follow-up visit and 709 (41.8%) were LTFU. Of the 1003 White patients, 597 (59.5%) attended their follow-up visit and 406 (40.5%) were LTFU. Of the 489 African American/Black patients, 267 (54.6%) attended their follow-up visit and 222 (45.4%) were LTFU. Of the 205 Other patients, 124 (60.5%) attended their follow-up visit and 81 (39.5%) were LTFU. Fig 1 illustrates the breakdown of patients that met the inclusion criteria and were analyzed in this study.

### Baseline characteristics

Baseline characteristics were identified for the total population that attended their follow-up visits and compared to those that were LTFU. Further, these same variables were analyzed for the White and African American/Black patients as depicted in Table 1. Three of 1,697 patients (0.2%), all of whom self-identified as Other race, did not have a specified diagnostic category. Two of these patients attended a follow-up visit, and the remaining one patient was LTFU.

**Table 1:** Baseline characteristics were identified for the total population and each cohort (White and African American/Black) that attended their follow-up visit and compared to those that were lost to follow-up (LTFU). Cohorts were analyzed and compared using two-tailed t-tests for continuous variables and Chi-square tests for categorical variables.

	All Patients N = 1697			White Patients N = 1003			African American/Black Patients N = 489			Comparison: White vs African American/Black Patients
Variable	Followed Up N = 988 (58.2%)	LTFU N = 709 (41.8%)	*P*-value	Followed Up N = 597 (59.5%)	LTFU N = 406 (40.5%)	*P*-value	Followed Up N = 267 (54.6%)	LTFU N = 222 (45.4%)	*P*-value	*P*-value
**Age (Years)**	48.9 ± 0.6	44.7 ± 0.7	<0.001	51.1 ± 0.8	48.4 ± 1.0	0.035	45.7 ± 1.0	39.2 ± 1.1	<0.0001	<0.0001
**Sex (Male)**	541 (54.8%)	430 (60.7%)	0.016	329 (55.1%)	244 (60.1%)	0.133	143 (53.6%)	127 (57.2%)	0.474	0.519
**Time Between Consultation and Follow-Up (Days)**	6.6 ± 0.3	10.5 ± 0.8	<0.001	7.0 ± 0.4	10.3 ± 0.9	0.001	6.4 ± 0.4	11.2 ± 1.8	0.009	0.834
**VA (LogMAR)**	0.73 ± 0.03	0.45 ± 0.03	<0.001	0.64 ± 0.03	0.41 ± 0.03	<0.001	0.84 ± 0.06	0.49 ± 0.05	<0.0001	0.006
**Distance to Outpatient Clinic (Miles)**	14.3 ± 0.5 N = 950	13.2 ± 0.6 N = 678	0.150	19.0 ± 0.7	17.8 ± 0.8	0.225	5.7 ± 0.4	6.0 ± 0.5	0.628	<0.0001
**High School Education (ZIP Code)**	88.2% N = 950	86.4% N = 678	<0.001	90.6%	89.5%	0.008	84.6%	82.7%	0.015	<0.0001
**Median Income (ZIP Code)**	46518 ± 601 N = 950	43135 ± 691 N = 678	<0.001	53883 ± 712	50685 ± 911	0.006	33399 ± 714	32774 ± 784	0.556	<0.0001
**Living Below Poverty Line (ZIP Code)**	19.1% N = 950	21.7% N = 678	<0.001	13.2%	15.2%	0.006	29.0%	30.6%	0.099	<0.0001
**Primary Language: English**	956 (96.8%)	682 (96.2%)	0.550	592 (99.2%)	401 (98.8%)	0.770	267 (100%)	222 (100%)	1.000	0.06
**Insurance: Medicaid**	153 (15.5%)	126 (17.8%)	0.200	70 (11.7%)	50 (12.3%)	0.854	61 (22.8%)	55 (24.8%)	0.695	<0.0001
**Number of Comorbidities**	0.41 ± 0.02	0.36 ± 0.03	0.130	0.36 ± 0.03	0.40 ± 0.04	0.329	0.52 ± 0.05	0.31 ± 0.05	0.003	0.25
**Trauma**	394 (39.9%)	368 (51.9%)	<0.001	240 (40.2%)	196 (48.3%)	0.012	101 (37.8%)	126 (56.8%)	<0.0001	0.283
**Cornea**	165 (16.7%)	75 (10.6%)	<0.001	109 (18.3%)	57 (14.0%)	0.097	40 (15.0%)	18 (8.1%)	0.026	0.023
**External Disease**	39 (3.9%)	40 (5.6%)	0.100	18 (3.0%)	21 (5.2%)	0.115	15 (5.6%)	15 (6.8%)	0.755	0.069
**Eyelid**	31 (3.1%)	32 (4.5%)	0.140	19 (3.2%)	16 (3.9%)	0.635	5 (1.9%)	11 (5.0%)	0.102	0.956
**Orbit**	16 (1.6%)	14 (2.0%)	0.580	7 (1.2%)	10 (2.5%)	0.190	7 (2.6%)	1 (0.5%)	0.124	1
**Pediatric**	3 (0.3%)	2 (0.3%)	0.670	3 (0.5%)	1 (0.2%)	0.905	0 (0%)	1 (0.5%)	0.929	0.897
**Glaucoma**	77 (7.8%)	15 (2.1%)	<0.001	40 (6.7%)	3 (0.7%)	<0.0001	26 (9.7%)	8 (3.6%)	0.013	0.038
**Neuro-ophthalmology**	62 (6.3%)	75 (10.6%)	0.0014	40 (6.7%)	46 (11.3%)	0.014	18 (6.7%)	20 (9.0%)	0.460	0.681
**Retina**	124 (12.6%)	67 (9.4%)	0.070	91 (15.2%)	48 (11.8%)	0.152	22 (8.2%)	13 (5.9%)	0.387	0.0002
**Uveitis**	75 (7.6%)	20 (2.8%)	<0.001	30 (5.0%)	7 (1.7%)	0.011	31 (11.6%)	9 (4.1%)	0.004	0.0004

### Logistic regression analyses

We performed logistic regression to identify variables associated with LTFU. Univariable logistic regression analysis was conducted on the overall patient population, as well as the White and African American/Black subgroups (Table 2). All variables with a p value < 0.15 were then used to generate a multivariable logistic regression model in a bi-directional stepwise fashion on overall patient population, White group and African American/Black group (Table 3).

**Table 2:** Univariable logistic regression analyses of factors associated with follow-up after eye-related consultations at a Level I trauma center. Odds ratios (ORs), 95% confidence intervals (CIs), and *p*-values are reported. An OR < 1.0 indicates increased odds of loss to follow-up (LTFU). Differences between White and African American/Black cohorts were assessed using Wald Z tests.

	All Patients, N = 1697			White Patients, N = 1003			African American/Black Patients, N = 489			Comparison
Variable	OR	95% CI	*P*-value	OR	95% CI	*P*-value	Odds Ratio	95% CI	*P-*value	*P-*value
**Age (per 10 years)**	1.12	1.06-1.19	<0.001	1.07	1.005-1.145	0.036	1.28	1.14-1.44	<0.0001	0.008
**Sex (Male)**	0.79	0.65-0.96	0.016	0.82	0.63-1.05	0.117	0.86	0.60-1.23	0.42	0.801
**Time Between Consultation and Follow-Up**	0.96	0.95-0.98	<0.001	0.98	0.96-0.99	0.0005	0.95	0.92-0.97	<0.0001	0.047
**Visual Acuity (LogMAR)**	1.56	1.37-1.79	<0.001	1.53	1.28-1.87	<0.0001	1.55	1.25-1.95	0.0001	0.958
**Distance to Outpatient Clinic (miles)**	1.01	1.00-1.01	0.15	1.01	1.00-1.01	0.226	0.99	0.97-1.02	0.619	0.402
**High School Education (ZIP Code)**	1.03	1.02-1.04	<0.001	1.03	1.01-1.05	0.007	1.03	1.01-1.06	0.019	0.985
**Median Income, Per $1,000 (ZIP Code)**	1.01	1.005-1.059	<0.001	1.01	1.003-1.018	0.006	1.005	0.989-1.021	0.555	0.5
**Living Below the Poverty Line (ZIP Code)**	0.99	0.98-0.99	0.001	0.98	0.97-0.99	0.005	0.99	0.97-1.00	0.097	0.801
**Primary Language: English**	0.85	0.50-1.46	0.55	0.68	0.19-2.45	0.54	NA	NA	NA	NA
**Insurance: Medicaid**	0.85	0.65-1.10	0.21	0.95	0.64-1.40	0.778	0.9	0.59-1.37	0.618	0.862
**Comorbidities**	1.11	0.97-1.26	0.14	0.92	0.77-1.09	0.326	1.44	1.13-1.86	0.004	0.003
**African American/Black vs White**	0.818	0.66-1.02	0.07	NA	NA	NA	NA	NA	NA	NA
**Other vs White**	1.04	0.77-1.42	0.8	NA	NA	NA	NA	NA	NA	NA
**Trauma**	0.62	0.51-0.75	<0.001	0.72	0.56-0.92	0.01	0.47	0.33-0.67	<0.0001	0.061
**Cornea**	1.7	1.27-2.28	<0.001	1.36	0.97-1.94	0.081	2.01	1.14-3.70	0.019	0.263
**External Disease**	0.69	0.44-1.08	0.1	0.57	0.30-1.08	0.085	0.83	0.39-1.75	0.617	0.452
**Eyelid**	0.69	0.41-1.14	0.14	0.8	0.40-1.59	0.517	0.37	0.11-1.03	0.068	0.232
**Orbit**	0.82	0.40-1.71	0.59	0.47	0.17-1.23	0.127	6	1.06-112.53	0.095	0.031
**Pediatric**	1.44	0.28-10.39	0.68	2.04	0.26-41.33	0.54	NA	NA	NA	NA
**Glaucoma**	3.97	2.33-7.22	<0.001	9.62	3.47-39.97	0.0002	2.91	1.35-7.00	0.01	0.102
**Neuro-ophthalmology**	0.56	0.40-0.80	0.002	0.56	0.36-0.87	0.011	0.74	0.38-1.43	0.365	0.503
**Retina**	1.34	0.98-1.84	0.071	1.34	0.92-1.96	0.128	1.46	0.72-3.04	0.3	0.837
**Uveitis**	2.83	1.75-4.80	<0.001	3.01	1.38-7.51	0.01	3.14	1.52-7.14	0.003	0.943

**Table 3:** Multivariable logistic regression analyses of factors associated with follow-up after eye-related consultations at a Level I trauma center. Odds ratios (ORs), 95% confidence intervals (CIs), and p-values are reported. An OR < 1.0 indicates increased odds of loss to follow-up (LTFU). All *p*-values < 0.05 were considered statistically significant.

	All Patients, N = 1697			White Patients, N = 1003			African American/Black Patients, N = 489		
Variable	OR	95% CI	P-value	OR	95% CI	P-value	Odds Ratio	95% CI	P-value
**Age (per 10 years)**	1.1	1.03-1.16	0.003	NA	NA	NA	1.26	1.12-1.43	0.004
**Sex (Male)**	0.76	0.61-0.95	0.014	NA	NA	NA	NA	NA	NA
**Time Between Consultation and Follow-Up**	0.97	0.96-0.98	<0.001	0.98	0.97-1.00	0.015	0.95	0.92-0.98	0.001
**Visual Acuity (LogMAR)**	1.36	1.17-1.58	<0.001	1.41	1.15-1.76	0.001	1.26	1.11-1.61	0.001
**High School Education (ZIP Code)**	1.02	1.00-1.05	0.035	1.03	1.01-1.06	0.005	1.03	1.01-1.06	0.019
**Median Income, per $1,000 (ZIP Code)**	1.01	0.99-1.02	0.46	NA	NA	NA	NA	NA	NA
**Living Below Poverty Line (ZIP Code)**	1	0.98-1.01	0.8	NA	NA	NA	NA	NA	NA
**Trauma**	0.79	0.61-1.01	0.064	NA	NA	NA	NA	NA	NA
**Cornea**	1.59	1.14-2.24	0.007	NA	NA	NA	2.15	1.10-4.38	0.029
**Glaucoma**	3.25	1.81-6.24	<0.001	7.54	2.64-31.77	0.001	NA	NA	NA
**Neuro-ophthalmology**	0.74	0.48-1.12	0.15	NA	NA	NA	NA	NA	NA
**Retina**	1.47	1.01-2.16	0.04	1.65	1.07-2.59	0.026	NA	NA	NA
**Orbit**	NA	NA	NA	NA	NA	NA	9.39	1.56-180.36	0.041
**Uveitis**	3.1	1.82-5.53	<0.001	2.71	1.20-6.93	0.024	3.45	1.52-8.68	0.005

On multivariable analysis, there was no statistically significant association between race and LTFU. In both the White and African American/Black subgroup analyses, residence in ZIP codes with lower rates of high school completion, a longer interval between the emergency department visit and the scheduled follow-up appointment, and better visual acuity at the time of consultation were independently associated with higher rates of LTFU. In both subgroup analyses, a diagnosis of uveitis was independently associated with lower rates of LTFU. Older age was independently associated with lower rates of LTFU in the overall cohort and in the African American/Black subgroup analysis. In the African American/Black subgroup analysis, orbital and corneal diagnoses were independently associated with lower rates of LTFU. In the White subgroup analysis, glaucomatous and retinal diagnoses were independently associated with lower rates of LTFU.

## Discussion

The current study identified several novel, independent predictors of LTFU after ophthalmology consultations at a level 1 trauma center, including predictors identified within race-stratified analyses of White and African American/Black patients. To minimize bias, improve consistency, and evaluate rates of LTFU in a race-agnostic manner, the current study incorporated multiple standardization strategies, including ZIP code-based proxies for social determinants of health and diagnosis standardization via the WEM [25,28–34].

Overall, the rate of LTFU did not differ significantly between White (40.5%) and African American/Black (45.4%) patients or between White and Other (39.5%) patients in multivariable analysis. Among White and African American/Black patients, several factors associated with LTFU were shared between the two groups. One of these was a diagnosis of uveitis, which was associated with a lower likelihood of LTFU in both groups. This association may reflect the clinical features of anterior uveitis, one of the most common diagnoses within this category, which often presents with ocular pain and may be associated with an underlying systemic disease. [36]. Prior studies have shown that pain can be a primary motivator for seeking medical care [37–39], suggesting that the symptomatic nature of uveitis may promote timely medical attention and continued follow-up. In contrast, absence of a high school diploma was associated with a higher rate of LTFU among both groups. Lower educational attainment has been associated with reduced health literacy, poorer health outcomes, and higher mortality [40–42]. Other studies have similarly highlighted the importance of health literacy and educational attainment in promoting overall health and adherence to recommended medical care, with evidence suggesting that educational attainment may have a greater influence on healthcare outcomes than factors such as income [43–45]. This is consistent with the findings of the current study, which demonstrated an independent association between lower high school graduation rates and LTFU, but not between income and LTFU.

Another factor associated with LTFU in both groups was visual acuity during the consultation, with better visual acuity being associated with a higher likelihood of LTFU. Near-normal visual acuity is generally associated with fewer symptoms and better health-related quality of life, although this relationship is complex and disease-dependent [46–48]. Consequently, patients experiencing a more severe decline in vision may perceive a greater need for continued ophthalmic care, providing one potential explanation for the lower rate of LTFU observed among those with worse visual acuity. An additional predictor of LTFU was the interval between the initial consultation and the scheduled follow-up appointment, with longer intervals being associated with a higher likelihood of LTFU. This relationship has been previously described [12,49] and likely stems from several mechanisms, including perceived urgency, forgetfulness, administrative barriers, and competing priorities [9,50].

Beyond the shared factors associated with LTFU, subgroup analyses identified unique factors associated with LTFU within each cohort. Corneal and orbital diseases, including conditions such as herpes simplex virus keratitis and inflammatory orbital disease, were associated with lower rates of LTFU in the African American/Black subgroup analysis. One possible explanation is that African American/Black patients may present at later stages of disease resulting in greater symptom burden at the time of evaluation, as has been reported in prior studies [51–53]. More severe and functionally disruptive symptoms associated with advanced disease, particularly pain, may increase the perceived urgency for care and improve follow-up adherence [51–53]. In addition, clinicians managing advanced disease may convey greater concern or urgency when discussing follow-up, further reinforcing adherence. Together, these findings raise the possibility that follow-up adherence may be influenced by a complex interplay of disease severity, symptom burden, patient perceptions, and clinician-directed follow-up recommendations.

Within the White cohort, glaucomatous and retinal diagnoses were associated with lower rates of LTFU. These associations were not statistically significant in the African American/Black subgroup analysis. Glaucomatous conditions, including acute angle-closure glaucoma, are comparatively more common in White patients and are typically painful and acutely vision-threatening [54–56]. In stark contrast, less painful and more chronic glaucomatous conditions, including open-angle glaucoma, disproportionately affect African American/Black patients [57]. Because these glaucomatous disease subtypes differ significantly in symptom burden and clinical urgency, variation in their distribution between patient populations may have contributed to the subgroup-specific follow-up patterns observed in the present study. The lower rate of LTFU observed with retinal disease may be explained somewhat differently. Acute retinal diseases are often painless at presentation despite causing substantial visual impairment [58–60]. As a result, perceived vision loss, rather than pain, may represent a stronger driver of follow-up adherence in these conditions, although this hypothesis was not directly evaluated in the present study. Health literacy may further influence patients’ responses to vision-threatening yet relatively painless disease, as prior studies have demonstrated associations between higher health literacy, greater educational attainment, and improved follow-up adherence [40,61,62]. Accordingly, the higher high school graduation rates observed among White patients in the present study may have contributed to the subgroup-specific follow-up patterns observed for glaucomatous and retinal diseases.

Beyond educational attainment, insurance status has also been associated with disparities in follow-up adherence, with prior studies reporting a significant relationship between Medicaid coverage and increased rates of LTFU [12,63,64]. This relationship, however, was not observed in the current study. Following ophthalmology consultations at ECMC, patients were scheduled for outpatient follow-up appointments at the REI and are provided up to two visits at no cost, irrespective of insurance status. Consequently, this practice may have reduced financial barriers to short-term follow-up and mitigated the impact of insurance status on follow-up adherence. This unique aspect of our institution's care model may, in part, explain why Medicaid coverage was not independently associated with LTFU in the present study, although the slightly lower proportion of Medicaid-insured patients in our cohort (17%) compared with the national average (24.2%) may have also influenced this finding [65].

Overall, the rate of LTFU observed in the present study was comparable to that reported in previous studies evaluating follow-up after eye-related ED visits [10,11,13,14]. One notable exception was a recent study conducted within the Yale-New Haven health system, which reported a LTFU rate of 25%, substantially lower than the 41.8% observed in the present study [12]. We hypothesize that this difference may be attributable to structural differences in the healthcare systems. ECMC and REI operate as separate entities with distinct contact numbers, administrative staff, and electronic medical record systems, which may complicate care coordination and the referral process. In contrast, Yale-New Haven operates within an integrated healthcare system, which may facilitate scheduling, communication, and continuity of care [12], a feature that likely streamlines the communication process and scheduling logistics between the ED clinics and patients. Moreover, the Yale-New Haven study also identified a correlation between having an established eye care provider and a higher likelihood of follow-up [12]. In the current study, patients were referred to their previously established outpatient providers outside of the REI when appropriate. Because these follow-up encounters were not captured in the primary analysis, the overall follow-up rate may have been underestimated relative to that reported in the Yale-New Haven study.

Several limitations should be considered when interpreting the findings of this study. First, the use of ZIP codes as a surrogate measure for socioeconomic variables provides an estimate rather than an exact measure of an individual patient's socioeconomic status [66,67]. Although several studies have demonstrated the utility of using zip codes as proxies to identify social determinants of health [28–34], an individual-level assessment of educational attainment and income would yield more nuanced insights into these critical factors. In addition, other unmeasured variables, including transportation access, appointment logistics, and other barriers to care, may have influenced follow-up adherence and should be considered when interpreting the present findings. Second, this study was conducted at a single safety-net hospital where follow-up visits are provided at no cost to patients. As a result, barriers related to healthcare access and financial burden may be reduced compared with other healthcare settings. Consequently, the findings may not be generalizable to healthcare systems where visit costs, insurance coverage, and other barriers to accessing care have a greater influence on follow-up adherence. Third, although our findings identify important associations with follow-up behavior, the retrospective design of this study does not permit determination of the underlying mechanisms driving these differences. Potential explanations, including differences in symptom burden, disease perception, health literacy, or other unmeasured factors, remain hypothesis-generating and warrant further investigation. Fourth, patients referred to outside providers were not tracked in the present study. Therefore, follow-up outcomes could not be ascertained for these patients, which may have resulted in misclassification of LTFU status. In addition, if referral patterns differed across patient subgroups, this limitation may have introduced selection bias and influenced the observed associations. Lastly, although analyses were performed separately by racial subgroup, formal interaction testing was not conducted. Therefore, subgroup-specific associations should be interpreted cautiously and not as evidence of differences between racial groups.

## Conclusions

The current study reports a LTFU rate of 41.8% following eye-related ED visits, with no significant difference in follow-up rates by race on multivariable analysis. Residence in ZIP codes with lower high school graduation rates, longer intervals between the ED visit and the scheduled follow-up appointment, and better presenting visual acuity were each independently associated with higher rates of LTFU in the overall cohort. Conversely, patients diagnosed with uveitis were less likely to be LTFU, suggesting that symptom severity and pain may influence follow-up adherence. Among African American/Black patients, corneal and orbital diseases were associated lower rate of LTFU. In contrast, White patients were less likely to be LTFU when presenting with glaucomatous and retinal diseases. These findings suggest that factors associated with LTFU may differ by racial subgroup and highlight opportunities for patient-centered interventions to reduce LTFU, which has been associated with worse visual outcomes and delayed management of systemic disease. Future prospective studies could further evaluate the impact of social determinants of health at a more granular level, including access to transportation, health literacy, and social support. This approach would offer a more comprehensive understanding of the barriers faced by patients and provide additional insight into strategies to effectively address them.

## Supporting information

S1 File Full list of diagnoses included in each diagnostic category. Diagnostic categories and corresponding diagnoses are based on The Wills Eye Manual: Office and Emergency Room Diagnosis and Treatment of Eye Disease [25].
(DOCX)

S2 File De-identified study dataset used for the analyses. The Microsoft Excel file contains the HIPAA-compliant de-identified data used for all analyses reported in this study.
(XLSX)
