# Supplementary material for: Determinants of loss to follow-up after eye-related emergency visits: A race-stratified analysis at a level I trauma center
Source: PLoS One. 2026 Aug 6;21(8):e0355731. doi: 10.1371/journal.pone.0355731 (PMC13446684; doi:10.1371/journal.pone.0355731)
Supplement: Supplementary file 1 [file pone.0355731.s001.docx]

| Category of Disease | Diagnosis |
| --- | --- |
| Trauma | Chemical Burn |
| Trauma | Corneal Abrasion |
| Trauma | Corneal and Conjunctival Foreign Body |
| Trauma | Conjunctival Laceration |
| Trauma | Traumatic Iritis |
| Trauma | Hyphemia and Microhyphema |
| Trauma | Iridodialysis/Cyclodialysis |
| Trauma | Eyelid Laceration |
| Trauma | Orbital Blowout Fracture |
| Trauma | Traumatic Retrobulbar Hemorrhage (Orbital Hemorrhage) |
| Trauma | Traumatic Optic Neuropathy |
| Trauma | Intraorbital Foreign Body |
| Trauma | Corneal Laceration |
| Trauma | Ruptured Globe and Penetrating Ocular Injury |
| Trauma | Intraocular Foreign Body |
| Trauma | Firework or Shrapnel-/Bullet-Related Injury |
| Trauma | Commotio Retinae |
| Trauma | Traumatic Choroidal Rupture |
| Trauma | Chorioretinitis Sclopetaria |
| Trauma | Purtscher Retinopathy |
| Trauma | Shaken Baby Syndrome |
| Cornea | Superficial Punctate Keratopathy |
| Cornea | Recurrent Corneal Abrasion |
| Cornea | Dry Eye Syndrome |
| Cornea | Filamentous Keratopathy |
| Cornea | Exposure Keratopathy |
| Cornea | Neurotrophic Keratopathy |
| Cornea | Ultraviolet Keratopathy |
| Cornea | Thygeson Superficial Keratopathy |
| Cornea | Pterygium/ Pinguecula |
| Cornea | Fungal Keratitis |
| Cornea | Acanthamoeba Keratitis |
| Cornea | Crystalline Keratopathy |
| Cornea | Herpes Simplex Virus |
| Cornea | Herpes Zoster Ophthalmicus/ Varicella Zoster Virus |
| Cornea | Interstitial Keratitis |
| Cornea | Staphylococcal Hypersensitivity |
| Cornea | Phlyctenulosis |
| Cornea | Contact Lens-Related Problems |
| Cornea | Contact Lens-Induced Giant Papillary Conjunctivitis |
| Cornea | Peripheral Corneal Thinning/ Ulceration |
| Cornea | Delle |
| Cornea | Keratoconus |
| Cornea | Fuchs Endothelial Dystrophy |
| Cornea | Aphakic Bullous Keratopathy/ Pseudophakic Bullous Keratopathy |
| Cornea | Corneal Graft Rejection |
| Cornea | Corneal Refractive Surgery Complications |
| Conjunctiva/ Sclera/Iris/External Disease | Acute Conjunctivitis |
| Conjunctiva/ Sclera/Iris/External Disease | Chronic Conjunctivitis |
| Conjunctiva/ Sclera/Iris/External Disease | Parinaud Oculoglandular Conjunctivitis |
| Conjunctiva/ Sclera/Iris/External Disease | Superior Limbic Keratoconjunctivitis |
| Conjunctiva/ Sclera/Iris/External Disease | Subconjunctival Hemorrhage |
| Conjunctiva/ Sclera/Iris/External Disease | Episcleritis |
| Conjunctiva/ Sclera/Iris/External Disease | Scleritis |
| Conjunctiva/ Sclera/Iris/External Disease | Blepharitis/Meibomitis |
| Conjunctiva/ Sclera/Iris/External Disease | Ocular Rosacea |
| Conjunctiva/ Sclera/Iris/External Disease | Mucous Membrane Pemphigoid (Ocular Cicatricial Pemphigoid) |
| Conjunctiva/ Sclera/Iris/External Disease | Contact Dermatitis |
| Conjunctiva/ Sclera/Iris/External Disease | Conjunctival Tumors |
| Conjunctiva/ Sclera/Iris/External Disease | Malignant Melanoma of the Iris |
| Eyelid | Ptosis |
| Eyelid | Chalazion/Hordeolum |
| Eyelid | Ectropion |
| Eyelid | Entropion |
| Eyelid | Trichiasis |
| Eyelid | Floppy Eyelid Syndrome |
| Eyelid | Blepharospasm |
| Eyelid | Canaliculitis |
| Eyelid | Dacryocystitis/Inflammation of the Lacrimal Sac |
| Eyelid | Preseptal Cellulitis |
| Eyelid | Malignant Tumors of the Eyelid |
| Orbit | Orbital Disease |
| Orbit | Inflammatory Orbital Disease |
| Orbit | Infectious Orbital Disease |
| Orbit | Orbital Tumors |
| Orbit | Traumatic Orbital Disease |
| Orbit | Lacrimal Gland Mass/Chronic Dacryoadenitis |
| Orbit | Miscellaneous Orbital Disease |
| Pediatrics | Leukocoria |
| Pediatrics | Retinopathy of Prematurity |
| Pediatrics | Familial Exudative Vitreoretinopathy |
| Pediatrics | Esodeviations |
| Pediatrics | Exodeviations |
| Pediatrics | Strabismus Syndromes |
| Pediatrics | Amblyopia |
| Pediatrics | Pediatric Cataract |
| Pediatrics | Ophthalmic Neonatorum (Newborn Conjunctivitis) |
| Pediatrics | Congenital Nasolacrimal Duct Obstruction |
| Pediatrics | Congenital/ Infantile Glaucoma |
| Pediatrics | Developmental Anterior Segment and Lens Anomalies/ Dysgenesis |
| Pediatrics | Congenital Ptosis |
| Pediatrics | The Bilaterally Blind Infant |
| Glaucoma | Primary Open-Angle Glaucoma |
| Glaucoma | Low-Tension Primary Open-Angle Glaucoma (Normal Pressure Glaucoma) |
| Glaucoma | Ocular Hypertension |
| Glaucoma | Acute Angle Closure Glaucoma |
| Glaucoma | Chronic Angle Closure Glaucoma |
| Glaucoma | Angle Recession Glaucoma |
| Glaucoma | Inflammatory Open-Angle Glaucoma |
| Glaucoma | Glaucomatocyclitic Crisis/Posner-Schlossman Syndrome |
| Glaucoma | Steroid-Response Glaucoma |
| Glaucoma | Pigment Dispersion Syndrome/ Pigmentary Glaucoma |
| Glaucoma | Pseudoexfoliation Syndrome/Exfoliative Glaucoma |
| Glaucoma | Lens-Related Glaucoma |
| Glaucoma | Plateau Iris |
| Glaucoma | Neovascular Glaucoma |
| Glaucoma | Iridocorneal Endothelial Syndrome |
| Glaucoma | Postoperative Glaucoma |
| Glaucoma | Aqueous Misdirection Syndrome/Malignant Glaucoma |
| Glaucoma | Postoperative Complications of Glaucoma Surgery |
| Glaucoma | Blebitis |
| Neuro-Ophthalmology | Anisocoria |
| Neuro-Ophthalmology | Horner Syndrome |
| Neuro-Ophthalmology | Argyll Robertson Pupil |
| Neuro-Ophthalmology | Adie (Tonic) Pupil |
| Neuro-Ophthalmology | Isolated Third Cranial Nerve Palsy |
| Neuro-Ophthalmology | Aberrant Regeneration of the Third Cranial Nerve |
| Neuro-Ophthalmology | Isolated Fourth Cranial Nerve Palsy |
| Neuro-Ophthalmology | Isolated Sixth Cranial Nerve Palsy |
| Neuro-Ophthalmology | Isolated Seventh Cranial Nerve Palsy |
| Neuro-Ophthalmology | Cavernous Sinus and Associated Syndromes (Multiple Ocular Motor Nerve Palsy) |
| Neuro-Ophthalmology | Myasthenia Gravis |
| Neuro-Ophthalmology | Chronic Progressive External Ophthalmoplegia |
| Neuro-Ophthalmology | Internuclear Ophthalmoplegia |
| Neuro-Ophthalmology | Optic Neuritis |
| Neuro-Ophthalmology | Papilledema |
| Neuro-Ophthalmology | Idiopathic Intracranial Hypertension/ Pseudotumor Cerebri |
| Neuro-Ophthalmology | Arteritic Ischemic Optic Neuropathy (Giant Cell Arteritis) |
| Neuro-Ophthalmology | Nonarteritic Ischemic Optic Neuropathy |
| Neuro-Ophthalmology | Posterior Ischemic Optic Neuropathy |
| Neuro-Ophthalmology | Miscellaneous Optic Neuropathies |
| Neuro-Ophthalmology | Nystagmus |
| Neuro-Ophthalmology | Transient Visual Loss/ Amaurosis Fugax |
| Neuro-Ophthalmology | Vertebrobasilar Artery Insufficiency |
| Neuro-Ophthalmology | Cortical Blindness |
| Neuro-Ophthalmology | Nonphysiologic Visual Loss |
| Neuro-Ophthalmology | Headache |
| Neuro-Ophthalmology | Migraine |
| Neuro-Ophthalmology | Cluster Headache |
| Retina | Posterior Vitreous Detachment |
| Retina | Retinal Break (Tear) |
| Retina | Retinal Detachment |
| Retina | Retinoschisis |
| Retina | Cotton-Wool Spot |
| Retina | Central Retinal Artery Occlusion |
| Retina | Branch Retinal Artery Occlusion |
| Retina | Central Retinal Vein Occlusion |
| Retina | Branch Retinal Vein Occlusion |
| Retina | Hypertensive Retinopathy |
| Retina | Ocular Ischemic Syndrome/Carotid Occlusive Disease |
| Retina | Diabetic Retinopathy |
| Retina | Vitreous Hemorrhage |
| Retina | Cystoid Macular Edema |
| Retina | Central Serous Chorioretinopathy |
| Retina | Nonexudative (Dry) Age-Related Macular Degeneration |
| Retina | Neovascular or Exudative (Wet) Age-Related Macular Degeneration |
| Retina | Idiopathic Polypoidal Choroidal Vasculopathy |
| Retina | Retinal Arterial Macroaneurysm |
| Retina | Sickle Cell Retinopathy |
| Retina | Valsalva Retinopathy |
| Retina | Pathologic/ Degenerative Myopia |
| Retina | Angioid Streaks |
| Retina | Ocular Histoplasmosis |
| Retina | Vitreomacular Adhesion/Vitreomacular Traction/Macular Hole |
| Retina | Epiretinal Membrane (Macular, Pucker, Surface-Wrinkling Retinopathy, Cellophane Maculopathy) |
| Retina | Choroidal Effusion/ Detachment |
| Retina | Retinitis Pigmentosa and Inherited Chorioretinal Dystrophies |
| Retina | Cone Dystrophies |
| Retina | Stargardt Disease (Fundus Flavimaculatus) |
| Retina | Best Disease (Vitelliform Macular Dystrophy) |
| Retina | Chloroquine/Hydroxychloroquine Toxicity |
| Retina | Crystalline Toxicity |
| Retina | Optic Pit |
| Retina | Solar or Photic Retinopathy |
| Retina | Choroidal Nevus and Malignant Melanoma of the Choroid |
| Uveitis | Anterior Uveitis (Iritis/Iridocyclitis) |
| Uveitis | Intermediate Uveitis |
| Uveitis | Posterior and Panuveitis |
| Uveitis | Human Leukocyte Antigen-B27-Associated Uveitis |
| Uveitis | Toxoplasmosis |
| Uveitis | Sarcoidosis |
| Uveitis | Behcet Disease |
| Uveitis | Acute Retinal Necrosis |
| Uveitis | Cytomegalovirus Retinitis |
| Uveitis | Noninfectious Retinal Microvasculopathy/HIV retinopathy |
| Uveitis | Vogt-Koyanagi-Harada Syndrome |
| Uveitis | Syphilis |
| Uveitis | Postoperative Endophthalmitis |
| Uveitis | Chronic Postoperative Uveitis |
| Uveitis | Traumatic Endophthalmitis |
| Uveitis | Endogenous Bacterial Endophthalmitis |
| Uveitis | Candida Retinitis/Endophthalmitis |
| Uveitis | Sympathetic Ophthalmia |

List of all categories of disease along with their corresponding diagnoses as used in the classification process in the current study. All categories were derived from *The Wills Eye Manual: Office and Emergency Room Diagnosis and Treatment of Eye Disease, 8th edition.^25^*
